# A Pilot Randomized Controlled Trial of Effect of Genioglossus Muscle Strengthening on Obstructive Sleep Apnea Outcomes

**DOI:** 10.3390/jcm10194554

**Published:** 2021-09-30

**Authors:** Maryam Maghsoudipour, Brandon Nokes, Naa-Oye Bosompra, Rachel Jen, Yanru Li, Stacie Moore, Pamela N. DeYoung, Janelle Fine, Bradley A. Edwards, Dillon Gilbertson, Robert Owens, Todd Morgan, Atul Malhotra

**Affiliations:** 1 Department of Medicine, University of California, La Jolla, San Diego, CA 92161, USA; mamaghsoudipour@health.ucsd.edu (M.M.); bnokes@health.ucsd.edu (B.N.); nabosompra@health.ucsd.edu (N.-O.B.); s3moore@health.ucsd.edu (S.M.); pdeyoung@health.ucsd.edu (P.N.D.); jfine@health.ucsd.edu (J.F.); dcgilbertson@health.ucsd.edu (D.G.); rowens@health.ucsd.edu (R.O.); 2 Department of Medicine, University of British Columbia, Vancouver, BC V6T 1Z4, Canada; rachjen@gmail.com; 3 Department of Otorhinolaryngology Head and Neck Surgery, Beijing Tongren Hospital, Capital Medical University, Beijing 100730, China; liyanruru@mail.ccmu.edu.cn; 4 Department of Physiology, School of Biomedical Sciences and Biomedical Discovery Institute, Monash University, Melbourne, VIC 3800, Australia; bradley.edwards@monash.edu; 5 Turner Institute for Brain and Mental Health, Monash University, Melbourne, VIC 3800, Australia; 6 Department of Dentistry, Scripps Encinitas Hospital, Encinitas, CA 92024, USA; todd@toddmorgan.com

**Keywords:** obstructive sleep apnea, adjunctive treatment, genioglossus muscle, continuous positive airway pressure, mandibular advancement splint

## Abstract

The genioglossus is a major upper airway dilator muscle. Our goal was to assess the efficacy of upper airway muscle training on Obstructive Sleep Apnea (OSA) as an adjunct treatment. Sixty-eight participants with OSA (AHI > 10/h) were recruited from our clinic. They fall into the following categories: (a) Treated with Automatic Positive Airway Pressure (APAP), (*n* = 21), (b) Previously failed APAP therapy (Untreated), (*n* = 25), (c) Treated with Mandibular Advancement Splint (MAS), (*n* = 22). All subjects were given a custom-made tongue strengthening device. We conducted a prospective, randomized, controlled study examining the effect of upper airway muscle training. In each subgroup, subjects were randomized to muscle training (volitional protrusion against resistance) or sham group (negligible resistance), with a 1:1 ratio over 3 months of treatment. In the baseline and the final visit, subjects completed home sleep apnea testing, Epworth Sleepiness Scale (ESS), Pittsburgh Sleep Quality Index (PSQI), SF-36 (36-Item Short Form Survey), and Psychomotor Vigilance Test (PVT). Intervention (muscle training) did not affect the AHI (Apnea-Hypopnea Index), (*p*-values > 0.05). Based on PSQI, ESS, SF-36 scores, and PVT parameters, the changes between the intervention and sham groups were not significant, and the changes were not associated with the type of treatment (*p*-value > 0.05). The effectiveness of upper airway muscle training exercise as an adjunct treatment requires further study.

## 1. Introduction

Obstructive sleep apnea (OSA) is defined by repetitive episodes of pharyngeal collapse during sleep [1,2]. OSA leads to excessive daytime sleepiness because of sleep fragmentation and other factors. Continuous positive airway pressure (CPAP) therapy reduces daytime sleepiness and the risk of cardiovascular morbidity and mortality, and it is known as the most effective intervention for sleep disordered breathing in severely affected patients [3,4]. Of note, incomplete adherence to CPAP treatment in some patients results in sub-optimal treatment outcomes. Oral appliances (mandibular advancement splints) are also considered to be a modality of treatment, but efficacy is variable and unpredictable [5,6].

The genioglossus muscle has a crucial role in the pathogenesis of OSA and is a major upper airway dilator. Studies evaluating genioglossus (GG) muscle activity at sleep onset suggest that patients with OSA have a marked reduction in activity in comparison with healthy individuals [7,8]. Mandibular advancement splints (MAS) pull the patient’s mandible in a forward and downward position to increase the airway patency in OSA patients [6,9].

The biomechanical behavior of the upper airway muscles is complicated [10,11]. Although using various modalities of increasing upper airway muscle tone has been controversial in the treatment of OSA [12], oropharyngeal exercises have shown promising results in some previous studies [13].

To our knowledge, there have been no controlled studies assessing the adjunct effect of tongue-muscle training on CPAP or MAS treatment, as a combination therapy. Therefore, we performed a randomized, double-blind, sham-controlled study to evaluate the efficacy of tongue-muscle training on Automatic Positive Airway Pressure (APAP) treatment, MAS treatment, or Untreated groups in OSA patients. We assessed the effectiveness of the intervention on the objective sleep measurements (e.g., polysomnography), as well as subjective sleep symptoms, including daytime sleepiness, sleep quality, and quality of life.

## 2. Materials and Methods

### 2.1. Participants

In this randomized clinical trial, patients previously diagnosed with obstructive sleep apnea (Apnea Hypopnea Index (AHI) > 10/h) were recruited from our sleep laboratory. The University of California San Diego Institutional Review Board approved all protocols and methods described adhered to the tenets of the Declaration of Helsinki and the Health Insurance Portability and Accountability Act. Written informed consents were obtained from all participants after the procedure had been explained. Our trial was registered on Clinical Trials (service of NIH): http://www.clinicaltrials.gov/NCT02502942 (accessed on 25 August 2021).

The inclusion criteria were the diagnosis of OSA with AHI > 10 events/h in patients 18–79 years of age. The exclusion criteria were patients with medically unstable status, pregnant women, current smokers, use of alcohol >3 oz/day or illicit drugs, consuming >10 cups of beverages with caffeine per day, and untreated sleep apnea with Epworth Sleepiness Scale (ESS) >18.

Participants were recruited from three subgroups of patients who were (a) Treated with APAP (*n* = 21), (b) Previously failed or refused CPAP therapy (Untreated), (*n* = 25), (c) Currently being treated with an oral appliance (MAS) who still have residual OSA (*n* = 22).

In the APAP group, participants were on APAP treatment for at least 3 months with good compliance (at least 4 h a day on average). In the “Untreated” group, untreated participants with OSA who have previously tried but were not currently using PAP therapy or an oral appliance. In “MAS” group, OSA patients had residual AHI > 10 events/h during oral appliance therapy. Participants in each group were randomized to upper airway muscle training group or sham group with ratio of 1:1 (35 patients received muscle training and 33 patients received a sham).

### 2.2. Procedure and Measurements

OSA patients who were interested in participating in our study were asked to review the informed consent at the sleep clinic for screening home sleep apnea testing (HSAT) to determine their eligibility. Our home sleep apnea test was Apnea Link (ResMed, Inc, San Diego, CA, USA). If they agreed to proceed and sign the informed consent for pre-screening HSAT, the patient was given a standard HSAT device with instructions to conduct one-night home sleep apnea testing. Apneas and hypopneas were defined according to the American Academy of Sleep Medicine (AASM) criteria [14]. Participant eligibility was determined based on their pre-screening HSAT results or a prior sleep study. If the patients were eligible, we then explained the study activities and obtained informed consent for the main study. The eligible patients had a known diagnosis of sleep apnea and were either untreated or on Automatic Positive Airway Pressure for at least 3 months or using Mandibular Advancement Splint (MSA) for at least 3 months.

At the baseline visit, the informed consent was obtained prior to the experimental visits. The anthropomorphic characteristics (height; weight; body mass index (BMI); neck, waist, and hip circumferences), sleep questionnaires (Epworth Sleepiness Scale (ESS) [15], Pittsburgh Sleep Quality Index (PSQI) [16], Short form 36 health survey questionnaire (SF-36) [17], and Psychomotor Vigilance Test (PVT) [18] were assessed at baseline and after 110 (±34) days of intervention.

The following OSA parameters were evaluated in first HSAT and the follow up HSAT for all patients: Apnea Hypopnea Index (AHI), Apnea Index (AI), Hypopnea Index (HI), and Oxygen Desaturation Index (ODI). Our primary outcomes defined in our clinical trial were changes in OSA, measured by AHI following intervention, and for the APAP group, changes in OSA pharyngeal mechanics as measured by change in the 95th percentile pressure level.

### 2.3. Upper Airway Muscle Training

#### 2.3.1. Pharyngeal Exercise Device

**Figure 1:**
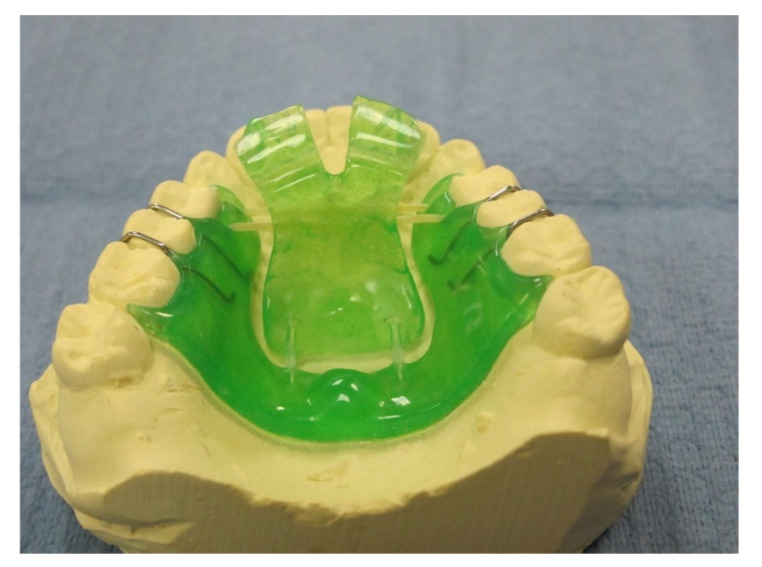
Oral muscle training device.

Following dental screening by a dentist, standard impressions were made for laboratory fabrication of a novel dental device that was designed to guide strength exercise to the lingual and pharyngeal muscles. The device is comprised of an acrylic-based plate worn on the palate, similar to a simple retainer, and secured to the upper arch using traditional orthodontic clasps, (Figure 1). The active device differs from the inactive device by having a hinge-related anterior palatal “flap” with orthodontic elastics, which provide resistance to pushing upwardly to contact the anterior portion of the palate. The control group was provided a sham device palatal plate without a hinge; they were told simply to clench on the occlusal acrylic periodically.

#### 2.3.2. Mode of Action (Intervention)

Depressing the hinge flap upward against the anterior palate for 10 min, twice a day, and meanwhile using 1–2 s compression bursts was one of the two active exercises. The second exercise required the participant to hold the flap up and then raise to posterior part of the tongue to reach a Target “bump” (shown) for a count of 2 s. In combination, these exercises engage the genioglossus muscle and then the lateral pharyngeal muscles, respectively [19].

### 2.4. Statistics

All statistical analysis was conducted at a confidence level of 95% using the software Stata version 15.0 (Stata Corp., College Station, TX, USA). We based power calculation on detecting a significant difference between AHI after intervention or sham. Assuming a final sample size of 34 patients in each group. We had an 80% power at the 0.05 significance level to detect a difference with an effect size as subtle as 0.7.

The distribution of numeric variables was assessed by inspecting histograms and using Shapiro–Wilk W tests of normality. Categorical variables were compared using the χ^2^ test. Test of significance was performed using Student’s *t*-test to compare the mean values of normally distributed variables: independent *t*-test for differences between the two study groups and paired *t*-test for changes of baseline to final IOP. Non-parametric tests such as Mann–Whitney U test and Wilcoxon signed-rank test were used whenever the variables were not normally distributed. The effect of the intervention on AHI, ODI, ESS subjective sleepiness ratings, PVT performance, and PSQI score were assessed with linear mixed model using time, intervention, and their interaction as factors. Subject ID was included as a random effect to account for individual differences. The models were also adjusted for age, gender, and treatment group and the effect of intervention in each treatment group was explored.

The models were refitted with possible confounders (that were borderline significant predictors (*p*-value < 0.1) of measurement magnitude in univariate models) to adjust for the effect of these variables.

## 3. Results

**Figure 2:**
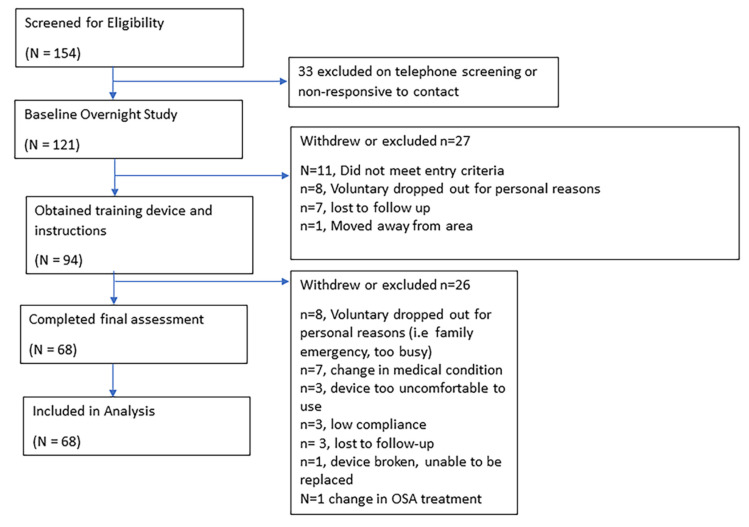
Enrollment flowchart.

From the 121 patients who were recruited initially, 68 patients were included in the final analysis (Figure 2). The demographic characteristics of the patients are presented in Table 1. In the sham group, the participants were significantly younger (63.2 ± 9.1 versus 56.0 ± 13.1 years, *p*-value, 0.038). The changes of snoring were not different between the intervention and sham groups (*p*-value, 0.505) (Table 1).

**Figure 3:**
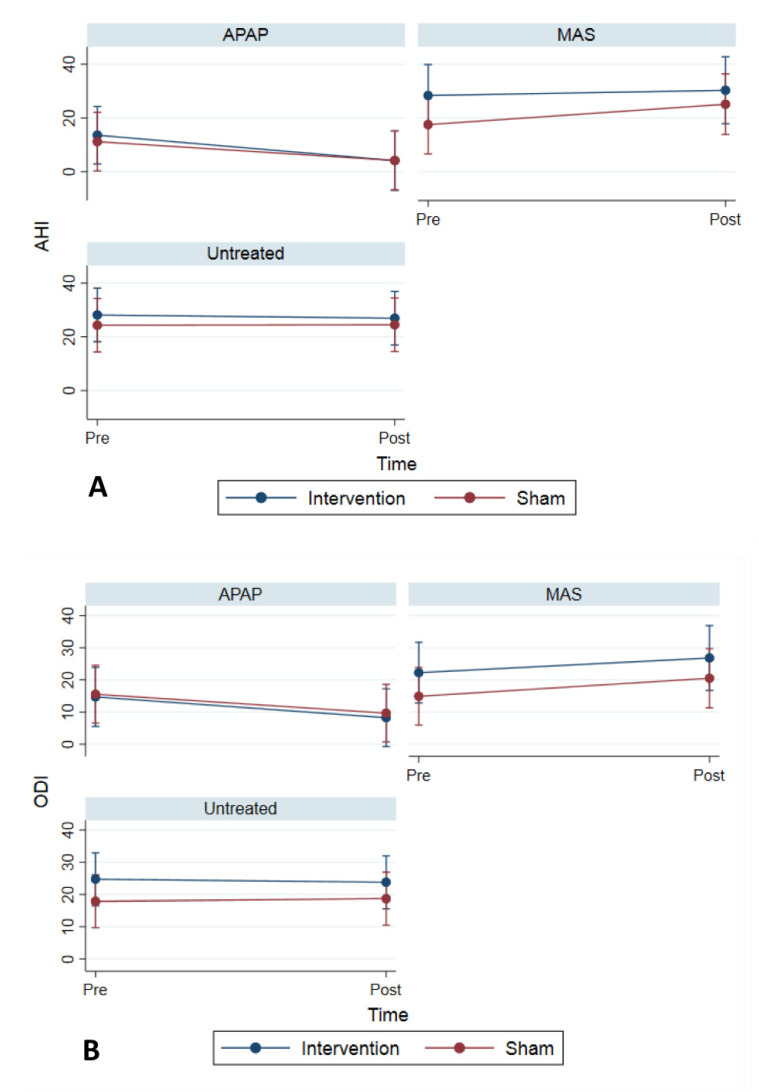
(**A**) Change in Apnea Hypopnea Index (AHI) across treatment groups. (**B**) Change in Oxygen Desaturation Index (ODI) across treatment groups.

Intervention (muscle training) did not affect the change in AHI, AI, and HI (Table 2), but the changes in AHI were different between the treatment groups (*p*-value, 0.006). A greater decrease in AHI was found in the APAP group compared to the MAS and Untreated groups (*p*-value, 0.023) (Figure 3A). Intervention (muscle training) did not affect the changes in the 95% APAP level (Table 2). Moreover, intervention (muscle training) was not associated with the changes in ODI (Table 2). The changes in ODI and OD-total were different among the treatment groups (*p*-value, 0.001 and 0.041, respectively), with a greater decrease in ODI and OD-total in the APAP group (Figure 3B). The results for the factors contributing to the change of AHI over time and tested in the multivariable mixed model are presented in Table 3.

**Figure 4:**
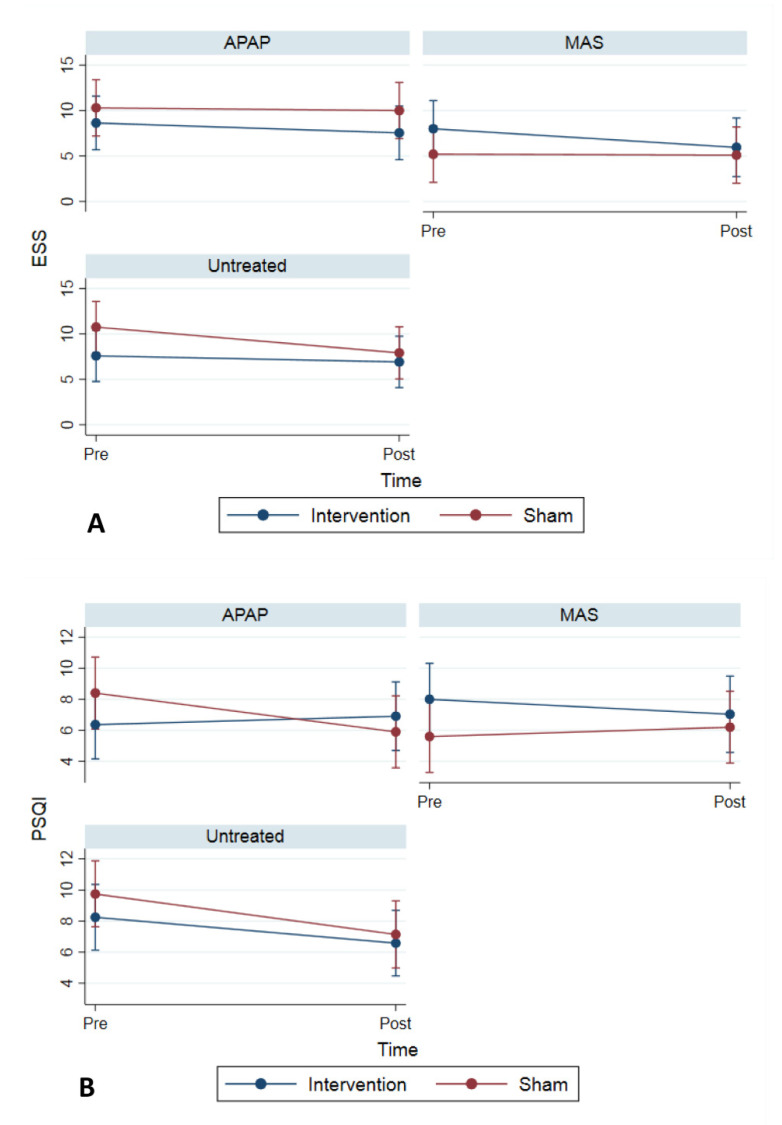
(**A**) Change in Epworth Sleepiness Scale (ESS) across treatment groups. (**B**) Change in Pittsburgh Sleep Quality Index (PSQI) across treatment groups.

ESS tended to decrease in both the intervention and sham groups (*p*-values, 0.072 and 0.084, respectively). However, the change was not significantly different between the intervention and sham groups (*p*-value, 0.397) (Table 4). The change in ESS was not different across the treatment groups (*p*-value, 0.850), (Figure 4A). The PSQI score in the sham group was significantly decreased (*p*-value, 0.004), but the changes between the intervention and sham groups were not significantly different (*p*-value, 0.056) (Table 4). While the change in the PSQI score was not different across the treatment groups (*p*-value, 0.590), in the APAP group, the decrease in the PSQI score was greater in the sham group compared to the intervention group (*p*-value, 0.022), (Figure 4B).

Generally, the PVT parameters improved at the final visit compared to the initial visit in both the intervention and sham groups. However, the changes between the intervention and sham groups were not significant (PVT_RT mean, PVT_slow10 mean, PVT lapses mean, and PVT false start); (*p*-values, 0.653, 0.058, 0.272, and 0.213, respectively) (Table 4). PVT lapses decreased significantly in the intervention group (*p*-value, 0.013). Improvements in PVT_RT mean, PVT_slow10 mean, PVT lapses mean, and PVT false start were not different among the treatment groups (*p*-values, 0.864, 0.894, 0.836, and 0.529, respectively). Changes in the PVT lapses were not different between the treatment groups.

In Table 5, the changes of subscales scores of the SF-36 questionnaires between initial and final visits are shown. “Energy/fatigue” increased significantly in the sham group (*p*-value, 0.037). Although “emotional well-being” increased significantly in the intervention group (*p*-value, 0.040), it was also increased in the sham group (*p*-value, 0.040). In addition, “Role limitations due to physical health” increased significantly in the sham group (*p*-value, 0.020) and the change between the intervention and sham groups was not significant (*p*-value, 0.078). However, none of the nine subscales show significant changes between the intervention and sham groups (Table 5).

## 4. Discussion

In the present study, pharyngeal muscle training was not associated with improvements in the objective and subjective sleep measurements in OSA patients. In the sham group, compared to the intervention group, the quality of life was decreased to a greater extent during the time of the follow-up period, demonstrated by increased role limitations and increased fatigue. Our results indicate that this particular training device was not effective for OSA treatment, and these results may inform future device designs as well as future studies regarding pharyngeal muscle training.

A systematic review evaluating new strategies targeted to increase upper airway patency in OSA patients assessed the studies that explored the effects of oropharyngeal exercises, as a complementary technique for treating OSA, and identified them to be effective, especially when the severity of the disease is moderate [2]. Although the effectiveness of hypoglossal nerve stimulation (HNS) is promising and has been accepted as a modality of treatment [20], the process is more invasive compared to oropharyngeal exercises. In turn, the attainment of more successful protocols of oropharyngeal exercises could be more beneficial.

The most extensive oropharyngeal exercises were described by Guimarães et al. In their study, patients had a significant decrease in neck circumference, snoring, daytime sleepiness, sleep quality, and OSA severity. The patients performed 30 min of daily exercise for 3 months [13]. Another study reported apnea-hypopnea index, snoring index, and minimum oxygen saturation improvements after oropharyngeal exercises in post-stroke apnea patients. Additionally, their exercise protocol improved subjective measurements of sleep quality, daily sleepiness, and performance [21]. The results of our study are in contrast with them, as we could not see any improvement in the apnea-hypopnea index and oxygen saturations. The different timing of therapy between the protocols and the specific muscles activated could be an explanation for the discrepancies.

In another study, the researchers instructed patients to perform oropharyngeal exercises three times a day, including six mastication patterns for approximately 8 min. Oropharyngeal exercises were effective in reducing objective measures such as snoring [19]. This was in contrast with our results, as we did not find any effects on snoring. Although their exercise protocol was similar to ours, their patients performed it three times a day in contrast with our twice a day protocol.

In a study assessing the effect of didgeridoo playing, daytime sleepiness and apnea-hypopnea index improved significantly. There was no effect on the quality of sleep and the health-related quality of life (SF-36) was not different between groups [22]. However, woodwind instrument methods may not be a fair comparison for isolated oropharyngeal training, given their concurrent role as a means of breathing exercise. One of the challenges in the treatment of OSA is poor compliance. In the present study, the exercises were selected based on previous studies, with a goal to improve the compliance [19]. During the experimental period, the subjects were assessed weekly to evaluate compliance. Convenience of use for the patient is a key factor for compliance. The other reason to choose this protocol with shorter time is that, in our study, the myofunctional therapy of oropharyngeal muscles was adopted as an adjunct therapy, and we tried to evaluate the combination of treatments. The device was built to mimic existing techniques deployed by Myo-functional therapy (MT), supported by previous studies [13,19,22].

In a recent meta-analysis evaluating the benefits of myofunctional therapy for the treatment of OSA, the authors concluded that myofunctional therapy may reduce daytime sleepiness and may increase sleep quality in the short term, and the certainty of the evidence ranges from moderate to very low, due to a lack of blinding, incomplete data and imprecision [23].

Although continuous positive airway pressure (CPAP) is considered the most efficient treatment for OSA [3], studies aiming to document the neurobehavioral outcomes of patients treated by CPAP have shown diverse results, and, of the SF-36 subscales, only the vitality subscale has shown significant improvement in more-adherent patients [24]. Patel and colleagues performed a meta-analysis showing that CPAP reduced the Epworth Sleepiness Scale (ESS) score in patients with OSA. The patients with moderate to severe OSA had a greater fall in ESS compared to those with mild OSA [25]. OSA severity might be a reason we did not see a significant effect of intervention in our patients, as most of our participants had mild or moderate OSA. Moreover, some studies suggest that OSA might lead to permanent structural brain abnormalities that contribute to neurobehavioral deficits in patients. Thus, cognitive symptoms and function may not be reversible with treatment, even if adherence is optimal [26]. This notion of irreversibility of some OSA consequences might be an explanation we could not see significant improvements of PVT parameters in the intervention group compared to the sham group.

Moreover, there is a study that has shown that the physiological traits that cause OSA also influence long-term CPAP adherence among those with OSA and coronary artery disease. A lower arousal threshold was associated with a marked reduction in CPAP use. Additionally, both high and low pharyngeal muscle compensation are linked to poor CPAP adherence. Therefore, identifying patients who are likely to benefit from genioglossus muscle strengthening, and future studies on more efficient genioglossus muscle strengthening protocols, might help the CPAP adherence in OSA patients [27].

Our study had certain limitations. First, the sample size was modest. Although we had a sufficient sample size for detecting the differences between the intervention and sham groups, we are not powered for the subgroups (APAP/MAS/Untreated). Second, our intervention was not universally tolerated, and our conclusions are limited to the population studied. Third, we did not examine dose–response relationships for the duration and frequency of pharyngeal muscle-training time in OSA patients as the intervention group only followed one protocol. Fourth, we acknowledge that most of our participants had mild or moderate OSA and patients with severe OSA may differ regarding the effect of genioglossus muscle strengthening on ESS or other parameters.

## 5. Conclusions

In the present study, we failed to prove the efficiency of upper airway muscle training exercise as an adjunct treatment in OSA. The exercise device might not adequately target the muscles important to airway patency. It is also possible that the dose (frequency, duration) of the exercise was not sufficient to strengthen the oropharyngeal muscles to reduce airway obstruction. Further research is recommended to determine the efficacy and the best modality for oropharyngeal muscle strengthening in OSA.

## Figures and Tables

**Table 1:** Characteristics of participants according to allocation to intervention and Sham groups.

Intervention/Sham (No.)	Intervention (*n* = 35) (Mean ± SD)	Sham (*n* = 33) (Mean ± SD)	*p*-Value
Age (mean ±SD)	63.2 ± 9.1	56.0 ± 13.1	0.038 *
Gender (M: F)	26:9	17:16	**0.052 ^†^**
Group of Treatment			
APAP	11	10	0.986 ^†^
MAS	11	11	
Untreated	13	12	
Initial BMI	30.0 ± 4.5	30.9 ± 7.1	0.930 *
Final BMI	30.1 ± 4.5	30.8 ± 6.8	0.979 *
*p* value of Change	0.681 ^‡^	0.750 ^‡^	0.615 ^§^
Initial neck circumference	40.1 ± 3.5	39.3 ± 4.5	0.411 *
Final neck circumference	40.3 ± 4.1	39.3 ± 4.5	0.823 *
*p* value of Change	0.431 ^‡^	0.975 ^‡^	0.674 ^§^
Initial Heart Rate	71.2 ± 14.5	72.1 ± 9	0.411 *
Final Heart Rate	68.3 ± 14.6	69.9 ± 12.5	0.823 *
*p* value of Change	0.591 ^‡^	**0.046 ^‡^**	0.935 ^§^
Initial Snoring (total number)	1056.4 ± 1093.7	1021.2 ± 1266.6	0.619 *
Final Snoring (total number)	1014 ± 1070.2	784.4 ± 1554.1	0.103 *
*p* value of Change	0.869 ^‡^	**0.028 ^‡^**	0.505 ^§^

* Student’s *t*-test or Mann–Whitney U test for normally or non-normally distributed variables, respectively. ^†^ Chi-squared test is used for categorized variable. ^‡^ Paired *t*-test or Wilcoxon signed-rank test for normally or non-normally distributed variables, respectively. ^§^ Linear mixed model. Bold fonts indicate significant differences.

**Table 2:** Polysomnography results in intervention and Sham groups.

Intervention/Sham	Intervention (*n* = 35) (Mean ±SD)	Sham (*n* = 33) (Mean ±SD)	*p*-Value
Initial AHI	23.8 ± 21.3	17.9 ± 17.6	0.250 *
Final AHI	19.9 ± 18.3	17.7 ± 16.2	0.611 *
Change	0.475 ^†^	0.728 ^†^	0.682 ^‡^
Initial AI	9.8 ± 13	5.5 ± 11.4	0.070 *
Final AI	8 ± 13.4	6.2 ± 9.3	0.865 *
Change	0.106 ^†^	0.585 ^†^	0.555 ^‡^
Initial HI	14 ± 13.1	12.4 ± 9.6	0.787 *
Final HI	11.9 ± 9.9	11.9 ± 11.7	0.621 *
Change	0.982 ^†^	0.522 ^†^	0.863 ^‡^
Initial AHI_4_	20 ± 14.8	19.6 ± 17.4	0.741 *
Final AHI_4_	17.9 ± 13.9	18 ± 12.8	0.844 *
Change	0.637 ^†^	0.820 ^†^	0.749 ^‡^
Initial ODI	20.7 ± 17.2	16 ± 13.1	0.401 *
Final ODI	18.1 ± 15.3	15.9 ± 12.3	0.788 *
Change	0.788 ^†^	0.788 ^†^	0.764 ^‡^
Initial OD total	150.7 ± 135.6	118.4 ± 109.1	0.455 *
Final OD total	129.5 ± 116.5	99.8 ± 78.6	0.674 *
Change	0.674 ^†^	0.506 ^†^	0.488 ^‡^
Initial APAP 95p	10.7 ± 2.6	11.9 ± 2.6	0.506 *
Final APAP 95p	10.5 ± 2.5	10.8 ± 2	0.772 *
change	0.593 ^†^	0.177 ^†^	0.649 ^§^

* Student’s *t*-test or Mann–Whitney U test for normally or non-normally distributed variables, respectively. ^†^ Paired *t*-test or Wilcoxon signed-rank test for normally or non-normally distributed variables, respectively. ^‡^ Linear mixed model adjusted for treatment, age, and gender. ^§^ Linear mixed model adjusted for age and gender.

**Table 3:** Factors Contributing to the Change of AHI over time by Mixed Model Analysis.

Variables	Univariable Model	
	β, 95% CI	*p*-Value
Age	−0.19 (−0.57, 0.19)	0.243
Gender: Female	−3.33 (−12.28, 5.63)	0.466
Group (baseline: Untreated)		
APAP	−5.11 (−14.65, 4.43)	0.294
MAS	7.97 (−1.72, 17.67)	0.107
Intervention (Muscle training)	−1.75 (−10.13, 6.63)	0.682

**Table 4:** Subjective sleep measurements and PVT (Psycho-motor Vigilance Test) results in intervention and Sham groups.

Intervention/Sham	Intervention (*n* = 35) (Mean ± SD)	Sham (*n* = 33) (Mean ± SD)	*p*-Value
Initial ESS Score	7.9 ± 5	8.8 ± 5.2	0.506 *
Final ESS Score	6.8 ± 4.4	7.5 ± 5.4	0.926 *
Change	0.072 ^†^	0.084 ^†^	0.397 ^‡^
Initial PSQI score	6.9 ± 3.5	8.1 ± 4	0.405 *
Final PSQI score	6.9 ± 3.5	6.4 ± 3.8	0.538 *
Change	0.476 ^†^	**0.004 ^†^**	**0.056 ^‡^**
Initial PVT_RT	334.4 ± 48.8	329.7 ± 43.1	0.689 *
Final PVT_RT	317.1 ± 36.2	310.5 ± 31.6	0.450 *
Change	**0.111 ^†^**	**0.003 ^†^**	0.653 ^‡^
Initial PVT_slow10	431.4 ± 44.6	426.6 ± 39.2	0.655 *
Final PVT_slow10	423.2 ± 32	402.2 ± 41.6	0.030 *
Change	0.413 ^†^	**0.003 ^†^**	**0.058 ^‡^**
Initial PVT lapses	3.8 ± 5.8	3.2 ± 5.7	0.640 *
Final PVT lapses	1.8 ± 2.9	1.3 ± 1.2	0.3051 *
Change	0.013 †	0.060 ^†^	0.272 ^‡^
Initial PVT false starts	0.5 ± 0.7	0.3 ± 0.4	0.220 *
Final PVT false starts	1 ± 1.1	0.4 ± 0.9	0.043 *
Change	0.003 ^†^	0.404 ^†^	0.213 ^‡^

* Student’s *t*-test or Mann–Whitney U test for normally or non-normally distributed variables, respectively. ^†^ Paired *t*-test or Wilcoxon signed-rank test for normally or non-normally distributed variables, respectively. ^‡^ Linear mixed model adjusted for treatment, age, and gender. Bold fonts indicate significant differences.

**Table 5:** Short form survey (SF-36) scoring results in intervention and Sham groups.

Intervention/Sham	Intervention (*n* = 35) (Mean ± SD)	Sham (*n* = 33) (Mean ± SD)	*p*-Value
Initial “Physical functioning”	76.6 ± 27.8	76.5 ± 24.2	0.673 *
Final “Physical functioning”	78.7 ± 23.7	79.2 ± 22.9	0.994 *
Change	0.370 ^†^	0.426 ^†^	0.457 ^‡^
Initial “Role limitations due to physical health”	75.7 ± 38.6	60.6 ± 42.9	0.130 *
Final “Role limitations due to physical health”	74.2 ± 41.7	79.7 ± 35	0.571 *
Change	>0.99 ^†^	**0.020 ^†^**	0.078 ^‡^
Initial “Role limitations due to emotional problems”	81 ± 35.5	71.7 ± 39.2	0.311 *
Final “Role limitations due to emotional problems”	85.9 ± 31.2	80.2 ± 33.7	0.486 *
Change	0.280 ^†^	0.324 ^†^	0.834 ^‡^
Initial “Energy/fatigue”	55.5 ± 20.9	48.7 ± 25.3	0.228 *
Final “Energy/fatigue”	57 ± 24.4	56.9 ± 24.3	0.987 *
Change	0.671 ^†^	**0.037 ^†^**	0.635 ^‡^
Initial “Emotional well-being”	73.1 ± 19.8	73.2 ± 18.3	0.993 *
Final “Emotional well-being”	80.2 ± 17.2	79.1 ± 16.5	0.790 *
Change	**0.040 ^†^**	**0.040 ^†^**	0.561 ^‡^
Initial “Social functioning”	80.7 ± 25.4	73.9 ± 27.5	0.294 *
Final “Social functioning”	83.3 ± 25.5	79.3 ± 25.9	0.527 *
Change	0.287 ^†^	0.120 ^†^	0.793 ^‡^
Initial “Pain”	72.9 ± 24.3	73.3 ± 24.5	0.990 *
Final “Pain”	74.3 ± 22.2	73.3 ± 26.4	0.884 *
Change	0.330 ^†^	0.620 ^†^	0.505 ^‡^
Initial “General health”	63.3 ± 21.5	64.2 ± 22.4	0.857 *
Final “General health”	63.6 ± 21.7	66.9 ± 21.3	0.538 *
Change	0.733 ^†^	0.388 ^†^	0.901 ^‡^
Initial “Health change”	57.1 ± 19.7	51.5 ± 22.5	0.275 *
Final “Health change”	57.6 ± 23	53.9 ± 22.1	0.514 *
Change	0.275 ^†^	0.441 ^†^	0.908 ^‡^

* Student’s *t*-test or Mann–Whitney U test for normally or non-normally distributed variables, respectively. ^†^ Paired *t*-test or Wilcoxon signed-rank test for normally or non-normally distributed variables, respectively. ^‡^ Linear mixed model adjusted for treatment, age, and gender. Bold fonts indicate significant differences.

## Data Availability

The data that support the findings of this study are available on request from the corresponding author. The data are not publicly available due to privacy or ethical restrictions.
